# Non-autonomous DAF-16/FOXO activity antagonizes age-related loss of *C. elegans* germline stem/progenitor cells

**DOI:** 10.1038/ncomms8107

**Published:** 2015-05-11

**Authors:** Zhao Qin, E. Jane Albert Hubbard

**Affiliations:** 1 Departments of Cell Biology and Pathology, The Helen L. and Martin S. Kimmel Center for Stem Cell Biology, Skirball Institute for Biomolecular Medicine, New York University School of Medicine, 540 First Avenue, New York, New York, USA

## Abstract

Stem cells maintain tissues and organs over the lifespan of individuals. How aging influences this process is unclear. Here we investigate the effects of aging on *C. elegans* germline stem/progenitor cells and show that the progenitor pool is depleted over time in a manner dependent on inhibition of DAF-16/FOXO by insulin/IGF-1 signalling (IIS). Our data indicate that DAF-16/FOXO activity in certain somatic gonad cells is required for germline progenitor maintenance, and that this role is separable from the effect of DAF-16/FOXO on organismal aging. In addition, blocking germ cell flux, similar to reducing IIS, maintains germline progenitors. This effect is partially dependent on gonadal DAF-16/FOXO activity. Our results imply that (1) longevity pathways can regulate aging stem cells through anatomically separable mechanisms, (2) stem cell maintenance is not necessarily prioritized and (3) stem cell regulation can occur at the level of an entire organ system such as the reproductive system.

## Abstract (web summary)

The number of germline stem/progenitor cells in *C. elegans* declines with age. Here the authors show this cell loss is mediated by the transcription factor DAF-16/FOXO acting in specific somatic gonad cells, demonstrating that stem cell aging can be anatomically uncoupled from organismal aging.

## Graphical abstract

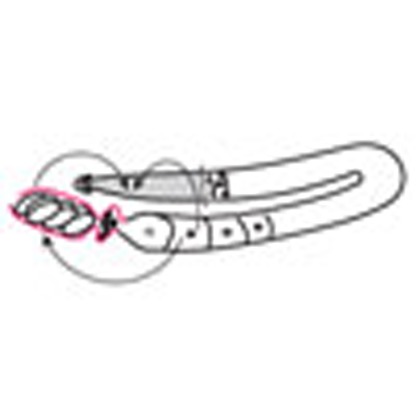

Stem cells are remarkable for their ability to maintain adult tissue homeostasis and to respond to injury. With age, however, stem cells experience numerical and/or functional decline or changes in differentiation potential, which can lead to bias in cancer predisposition, tissue degeneration and increased susceptibility to tissue damage^1^. Therefore, a better understanding of how aging affects stem cells may provide important insights relevant to age-related diseases and stem cell-based therapy.

The nematode *C. elegans* provides an attractive model for studying stem cell aging. First, *C. elegans* possesses a relatively simple and accessible stem cell system—the germline stem cells—that, similar to stem cell systems in other organisms, uses the conserved Notch signalling pathway as the major pathway to maintain stem cell fate^2^,^3^ (Fig. 1a). Second, *C. elegans* is a well-established genetic model for aging. The relatively short (2–3 weeks) lifespan of laboratory worms facilitates the analysis of age-dependent events. Many of the longevity pathways initially identified in worms are highly conserved for aging functions across species^4^.

Here we investigated the effects of age on the pool of undifferentiated proliferative cells in the distal end of the *C. elegans* germ line. This population of cells includes both germline stem cells and their proliferative progeny (hereafter referred to as ‘germline progenitor cells', as no markers currently distinguish stem cells from their proliferative progeny; Fig. 1a). We observed a marked age-dependent decline in the number of germline progenitor cells that is far less severe in mutants with reduced insulin/insulin-like growth factor-1 (IGF-1) signalling (IIS). In addition, we found that DAF-16/FOXO acts downstream of IIS to regulate germline progenitor maintenance. By modulating DAF-16/FOXO activity in a tissue-specific manner, we found that the extent of germline progenitor loss over time could be uncoupled from the rate of organismal aging. Surprisingly, DAF-16/FOXO activity is not required in germ cells, but rather is required in somatic cells of the gonad to prevent germline progenitor cell loss. Specifically, DAF-16/FOXO activity is required at the proximal end of the reproductive tract, in cells that contact transiting gametes and embryos. Finally, we determined that germ cell flux also influences germline progenitor maintenance through DAF-16/FOXO-dependent and DAF-16/FOXO-independent mechanisms.

## Results

### The number of germline progenitors decreases with age

**Figure 1:**
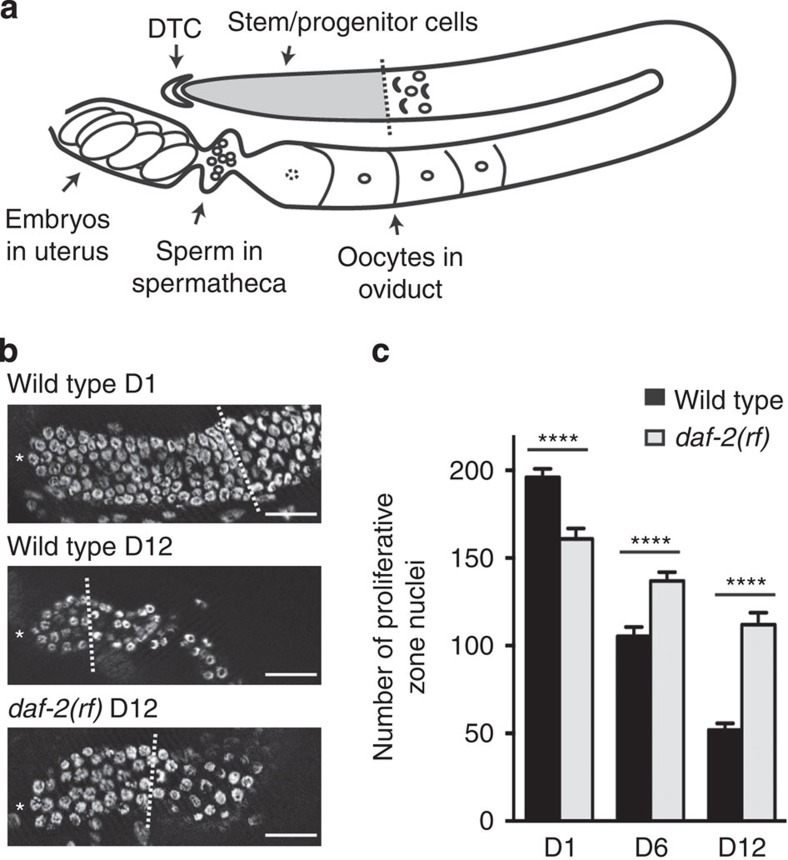
The DAF-2/insulin-IGF-like receptor (IIR) promotes age-related loss of germline progenitor cells. (**a**) Schematic drawing of the adult *C. elegans* hermaphrodite germ line. Stem/progenitor cells (grey shading) are located in the distal region of the germ line that is capped by the distal tip cell (DTC), the stem cell niche. As germ cells divide and move proximally, they enter meiosis. Nuclei in early stages of prophase of meiosis I (leptotene and zygotene) are crescent shaped. By convention, the appearance of two or more crescents in a full ring of nuclei marks the proximal border of the progenitor pool^37^ (dotted line). Germ cells eventually differentiate into sperm that are stored in the spermatheca followed by oocytes that reside in the adult oviduct. In response to signals from the sperm, oocytes mature one by one and are fertilized as they pass through the spermatheca. (**b**) Representative DAPI-stained wild-type and *daf-2(rf)* germ lines. Asterisk indicates the distal end of the germ line, and the dotted line indicates the proximal boarder of the proliferative zone. Scale bars, 20 μm. (**c**) Time course of germline progenitor depletion in wild-type and *daf-2(rf)* animals. Note that *daf-2(rf)* mutants start with fewer germline progenitor cells than wild type on adult day 1 (D1) (see text for details). Error bar indicates s.e.m.; ****P<0.0001 by two-tailed Student's *t*-test; also, pairwise comparisons within genotypes for each time point *P*<0.0001 by Student's *t*-test. Allele used: *daf-2(e1370)*. See Supplementary Table 3 for complete data. DAPI, 4,6-diamidino-2-phenylindole.

We determined the number of progenitor cells in the germline proliferative zone of wild-type *C. elegans* hermaphrodites during adulthood and found that the pool of germline progenitors decreased markedly over time. Under normal laboratory growth conditions, germline progenitors accumulate during larval development to a pool of ∼200–250 cells at early adulthood^5^. This pool is maintained for 24–36 h, but decreases thereafter^5^,^6^,^7^. We found that by 12 days of adulthood, the progenitor pool was reduced to ∼50 cells in the wild type (Fig. 1b,c).

We considered three possible cellular mechanisms for the loss of germline progenitors in aged worms as follows: (1) cell death, (2) a cell cycle defect and/or (3) a change in the balance between proliferation (mitosis) and differentiation (meiosis), such that meiotic entry occurred more distally in older animals. For cell death, we examined the *ced-3*/Caspase mutant, as germ cell death in *C. elegans* is dependent on the core apoptotic machinery^8^. We found that although clusters of extra germ cells accumulated in the pachytene stage in old *ced-3(−)* animals (Supplementary Fig. 1a), the number of germline progenitor cells was not significantly altered (Supplementary Fig. 1b), suggesting that inappropriate progenitor apoptosis does not account for germline progenitor loss over time. To assess cell cycle, we measured the mitotic index in the proliferative zone and found that the mitotic index of day 12 (D12) animals was reduced relative to that of D1 animals (Supplementary Fig. 1c), suggesting a role for cell cycle in progenitor loss. For the mitosis versus meiosis cell fate decision, we determined the distance from the distal tip to the proximal border of the proliferative zone as a measure of the effective ‘reach' of the distal tip cell signal to deter meiotic entry. We found that the distance to meiotic entry was reduced in D12 animals compared with D1 animals (Supplementary Fig. 1d), indicative of an additional role for cell fate decision in this phenotype. Notch signalling mediated by the Notch family receptor GLP-1 controls the cell fate decision in the *C. elegans* germ line^2^,^9^. Consistent with a prominent cell fate role, the combination of aging and reduced *glp-1* activity enhanced the penetrance of the ‘all meiotic' Glp-1 phenotype (in which all germ cells enter meiosis and the progenitor pool is lost). For example, in contrast to the wild type that retained ∼100 progenitor cells on D6, 30% of *glp-1-reduction-of-function (rf)* mutant animals had the ‘all meiotic' phenotype. By D12, nearly all *glp-1(rf)* animals failed to maintain a progenitor pool (Supplementary Fig. 1e). Together, we conclude that both reduced cell division rate and altered cell fate, but not cell death, contribute to the loss of germline progenitors over time.

### DAF-2/IIR promotes age-related loss of germline progenitors

Because the germline progenitor cell loss phenotype is age related, we asked whether genes that affect the rate of aging would influence germline progenitor maintenance over time. Reducing the highly conserved IIS delays aging and extends lifespan in many organisms^4^. Worms with reduced activity of the sole insulin/IGF-like receptor (IIR) in *C. elegans*, *daf-2*, live twice as long as wild type^10^. In addition, some *daf-2(rf)* mutants have extended reproductive span and produce progeny late in life^11^,^12^. Therefore, we extended previous studies^7^ and examined the D1–D12 time course of germline progenitor cell depletion in two *daf-2(rf)* mutants, *daf-2(e1370)* and *daf-2(e1368)*. Due to a requirement for IIS in the accumulation of larval germline progenitors, *daf-2(rf)* animals start adulthood with fewer germline progenitors^13^ (D1, Fig. 1c and Supplementary Fig. 2). Despite this initial shortfall, both mutants maintained more progenitor cells on D6 and D12 compared with wild type (Fig. 1b,c; Supplementary Fig. 2). The maintenance is more pronounced in *daf-2(e1370)* in which D12 animals retained an average of twice as many progenitors as wild type (112.0±6.8 versus 52.0±3.7), and this allele was used in further analysis. We conclude that DAF-2/IIR signalling promotes age-related loss of germline progenitors.

### DAF-16/FOXO acts downstream of DAF-2/IIR

**Figure 2:**
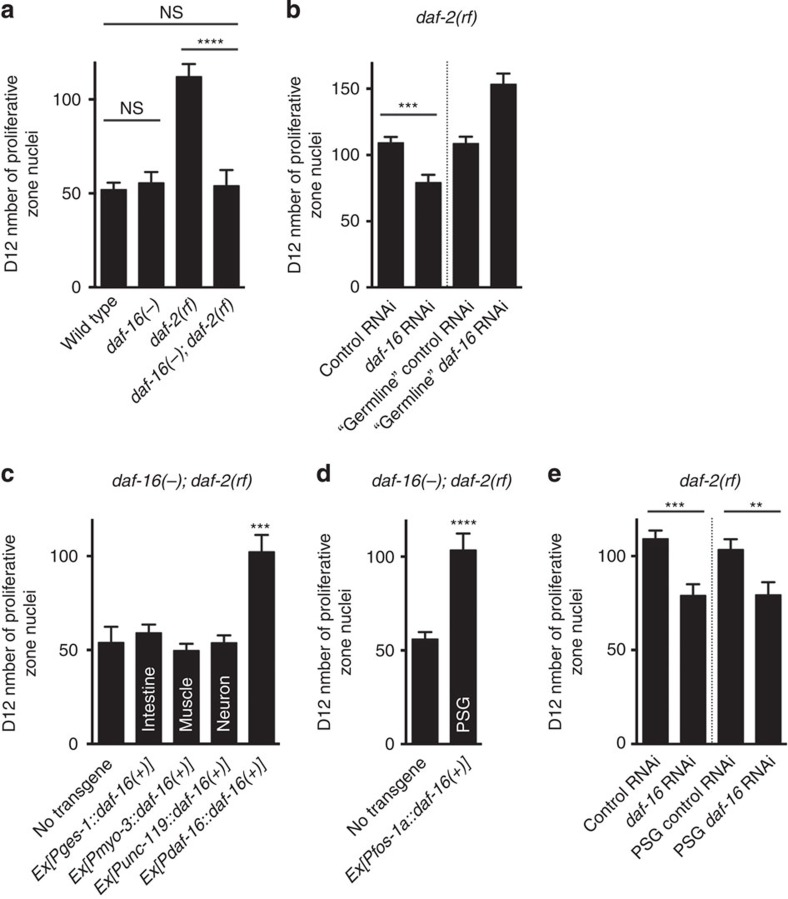
Proximal somatic gonad (PSG) DAF-16/FOXO activity maintains germline progenitors. Average number of proliferative zone nuclei in D12 (**a**) wild-type, *daf-16(−)*, *daf-2(rf)* and *daf-16(−); daf-2(rf)* animals, (**b**) *daf-2(rf)* and *rrf-1(−); daf-2(rf)* animals treated with control and *daf-16* RNAi, (**c**) *daf-16(−); daf-2(rf)* and *daf-16(−); daf-2(rf)* animals carrying a transgene expressing *gfp::daf-16(+)* from the *ges-1*, *myo-3*, *unc-119* and *daf-16* promoters, respectively, (**d**) *daf-16(−); daf-2(rf)* and *daf-16(−); daf-2(rf)* animals carrying a transgene expressing *gfp::daf-16(+)* from the *fos-1a* promoter, (**e**) *daf-2(rf)* and *daf-2(rf); rde-1(−); Is[Pfos-1a::rde-1(+)]* animals treated with control and *daf-16* RNAi. (**b**) Note that due to the germline-autonomous requirement for *daf-2* in larval progenitor accumulation (which is *daf-16* dependent), ‘germline' *daf-16* RNAi animals start with more progenitors on D1 and, therefore, are left with more germline progenitors on D12 than the RNAi control (see Supplementary Fig. 3). The decline between D1 and D12 is similar for ‘germline' control and *daf-16* RNAi. (**c**,**d**) In all cases, *daf-16(+)* encodes DAF-16 isoform a1. Label on the bar indicates the tissue that expresses the transgene. Error bar indicates s.e.m.; NS, *P*>0.05; **P<0.01; ***P<0.001; ****P<0.0001 by two-tailed Student's *t*-test. Alleles used: *daf-2(e1370), daf-16(mu86)* (in **a**,**c**), *daf-16(m26)* (in **d**), *rrf-1(pk1417)* and *rde-1(ne219)*. See Supplementary Table 3 for complete genotypes and data. NS, not significant.

IIS controls many processes in *C. elegans*, including metabolism, immunity, development and aging. In most cases, activation of the receptor, DAF-2, initiates a conserved kinase cascade that ultimately inhibits the FOXO transcription factor DAF-16 (ref. 14). To determine whether *daf-16* mediates the effect of IIS on germline progenitor cell maintenance, we asked whether, similar to other phenotypic scenarios, the effect of reduced *daf-2* activity could be suppressed by the loss of *daf-16*. We found that while loss of *daf-16* alone did not affect germline progenitor loss over time compared with wild type (Fig. 2a, *daf-16(−)* versus wild type), in the absence of *daf-16*, *daf-2(rf)* animals lost their germline progenitors to the same extent as the wild type (Fig. 2a, *daf-16(−); daf-2(rf)* versus *daf-2(rf)* and wild type, respectively). Therefore, *daf-16(+)* activity is required for the ability of *daf-2(rf)* to maintain germline progenitors over time.

### DAF-16/FOXO acts in the proximal somatic gonad

To determine whether *daf-16(+)* maintains germline progenitors in a germline-autonomous manner, we tested the effect of knocking down *daf-16* by RNA interference (RNAi) in the *daf-2(rf)* background in the presence and absence of *rrf-1*. In the absence of *rrf-1*, RNAi effectiveness is reduced in the soma but retained in the germ line^15^,^16^. We found that while *daf-2(rf)* animals subject to *daf-16* RNAi resembled the *daf-16(−); daf-2(rf)* double mutants and lost their germline progenitors similarly to wild type, this effect was not observed in the *rrf-1(−); daf-2(rf)* animals subject to *daf-16* RNAi (Fig. 2b). However, *daf-16* RNAi was indeed effective in the *rrf-1(−); daf-2(rf)* background, as evidenced by suppression of the germline-autonomous larval germline proliferation phenotype caused by the *daf-2(rf)* mutation (Supplementary Fig. 3), consistent with previous results^13^. These results suggest that *daf-16(+)* is not required in the germ line for *daf-2(rf)* to preserve germline progenitors over time.

To determine the anatomical focus of *daf-16(+)* activity for germline progenitor maintenance, we employed two strategies as follows: (1) tissue-specific expression of a rescuing *daf-16(+)* transgene and (2) tissue-specific *daf-16* RNAi. Surprisingly, we found that *daf-16(+)* expression in a subset of somatic cells in the proximal gonad is sufficient to maintain germline progenitors. First, expression of a *gfp::daf-16* transgene from the *fos-1a* promoter in a subset of adult spermathecal and uterine cells^17^ (Supplementary Fig. 4a) restored the number of D12 progenitors in the *daf-16(−); daf-2(rf)* double mutant background to the level seen in *daf-16(+); daf-2(rf)* animals and to the level seen in *daf-16(−); daf-2(rf)* animals expressing the same *gfp::daf-16* transgene from the *daf-16* endogenous promoter (Fig. 2c,d). By contrast, expression of the same *gfp::daf-16* transgene in the intestine, muscles or neurons did not affect germline progenitor cell maintenance (Fig. 2c). We verified both *daf-16(+)* activity by rescue of relevant phenotypes and the corresponding tissue-specific *daf-16* transgene expression in aged animals, as previously reported^18^. The same negative result was observed with a *daf-16::gfp* transgene expressed in the distal tip cell^13^, the stem cell niche (Supplementary Fig. 5a).

We confirmed the proximal somatic gonad requirement for *daf-16(+)* in germline progenitor maintenance by tissue-specific *daf-16* RNAi. To achieve proximal somatic gonad-specific RNAi, we performed RNAi knockdown in the *rde-1(−)* RNAi-deficient animals expressing *rde-1(+)* from the *fos-1a* promoter^19^. We found that knocking down *daf-16* by proximal somatic gonad-directed RNAi resembled *daf-16* RNAi in the whole animal, causing more pronounced germline progenitor depletion in the *daf-2(rf)* background (Fig. 2e).

Since expression of *gfp::daf-16* in the intestine has a marked effect on lifespan^18^, failure of the same transgene to influence germline progenitor cell maintenance (Fig. 2c) suggests that DAF-16/FOXO regulates lifespan and germline progenitor maintenance separately. Another tissue that has been implicated in lifespan regulation is the hypodermis^20^. To test whether hypodermal IIS influences germline progenitor maintenance, we performed *daf-2* RNAi in *rde-1(−)* animals expressing *rde-1(+)* from a hypodermis-specific version of the *lin-26* promoter^21^. We found that hypodermis-specific *daf-2* knockdown had no effect on progenitor maintenance, although *daf-2* RNAi in the whole animal or in the proximal somatic gonad alone could mimic the effect of the *daf-2(rf)* mutation, maintaining more germline progenitors than the RNAi control on D12 (Supplementary Fig. 5b,c).

To further test the hypothesis that lifespan and germline progenitor cell maintenance are independent phenotypes that can be uncoupled anatomically, we asked whether the same proximal somatic gonad-expressing *gfp::daf-16* transgene that was sufficient for germline progenitor maintenance would influence lifespan. We found a minimal difference between the lifespans of proximal somatic gonad *daf-16(+)* animals and their non-transgenic siblings: 16.1±0.5 days and 14.8±0.4 days, respectively (*P*=0.04; Supplementary Fig. 4b).

Together, our data reveal a novel cell non-autonomous DAF-16/FOXO activity that is required for adult germline progenitor maintenance and that is anatomically separable from its previously described roles in lifespan regulation and other biological processes in *C. elegans*.

### Germ cell flux promotes germline progenitor cell loss

The proximal somatic gonad is a region of the reproductive tract that contacts transiting gametes and embryos. Our finding that DAF-16/FOXO is required in this region suggested the possibility that germ cell flux through the proximal reproductive tract might influence the distal germline progenitor cell pool. We took advantage of *C. elegans* reproductive biology to test this hypothesis. The hermaphrodite reproductive system is essentially female except that ∼300 self-sperm are produced before exclusive oocyte production^22^. Since hermaphrodites produce oocytes in excess, the number of self-progeny produced by unmated hermaphrodites is limited by the number of self-sperm, and reproduction ends when self-sperm are depleted. By contrast, when provided with additional sperm through mating, hermaphrodites can produce as many as 1,000 progeny and their reproductive span is extended^23^.

**Figure 3:**
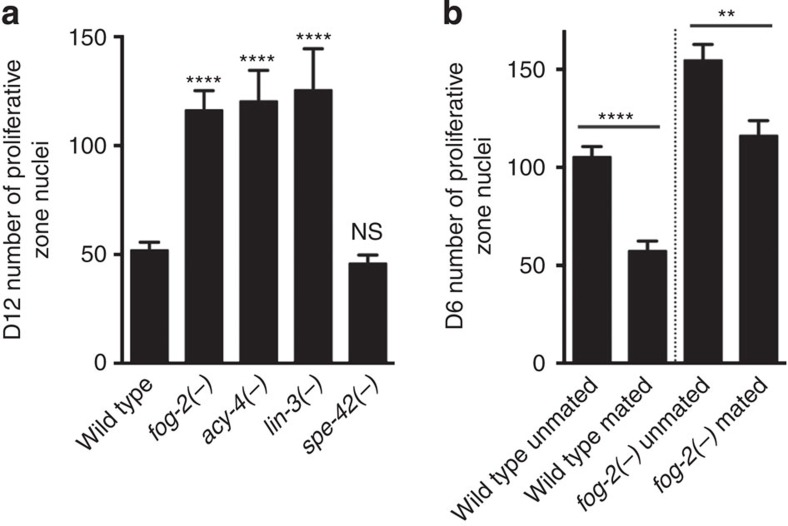
Germ cell flux promotes germline progenitor loss. Average number of proliferative zone nuclei in (**a**) D12 wild-type, *fog-2(−)*, *acy-4(−)*, *lin-3(−)* and *spe-42(−)* animals, (**b**) D6 wild type and *fog-2(−)* mutants that are unmated or have been mated with young, wild-type adult males since D4. Error bar indicates s.e.m.; NS, *P*>0.05; **P<0.01; ****P<0.0001 by two-tailed Student's *t*-test. Alleles used are as follows: *fog-2(oz40)*, *acy-4(ok1806)*, *lin-3(n1058)* and *spe-42(tn1231)*. See Supplementary Table 3 for complete genotypes and data.

We, therefore, tested the hypothesis that germ cell flux might influence the distal germline progenitor pool by manipulating sperm availability. First, we examined *fog-2(−)* females that produce no self-sperm. These animals ovulate at a reduced rate due to the lack of the major sperm protein oocyte maturation signal from sperm, and, thus, have a much slower flux of germ cells through their reproductive tract. As a result, un-ovulated immature oocytes stack up in the oviduct^24^. We found that *fog-2(−)* females contained many more germline progenitor cells than wild-type hermaphrodites on D12 (Fig. 3a), suggesting that blocking germ cell flux inhibits the loss of germline progenitors over time. To test whether increasing germ cell flux would have the opposite effect, we mated young, wild-type adult males with wild-type hermaphrodites or *fog-2(−)* females on D4, when wild-type hermaphrodites have almost exhausted their self-sperm^12^. By D6, both mated wild-type hermaphrodites and *fog-2(−)* females had fewer germline progenitors relative to their unmated controls, respectively (Fig. 3b), again supporting the notion that germ cell flux promotes germline progenitor cell loss and that germline progenitors are essentially ‘used up' over time.

To further investigate a correlation between germline progenitor loss and germ cell flux, we examined two additional mutants, *acy-4(−)* and *lin-3(−)*, in which germ cell flux is blocked in the presence of self-sperm. These mutations interfere with oocyte maturation and ovulation, respectively^25^,^26^. We found that similar to *fog-2(−)*, germline progenitor cell loss is inhibited in animals bearing either of these two mutations (Fig. 3a). Because reducing germ cell flux inhibits germline progenitor cell loss independent of the presence of sperm, our results suggest that germ cell flux, rather than sperm availability, influences germline progenitor maintenance over time.

To rule out a role for fertilization and progeny production in germline progenitor maintenance, we tested a fertilization-defective mutant, *spe-42(−)*. In this mutant, sperm are present and produce major sperm protein that promotes oocyte meiotic maturation, but these sperm are incapable of fertilizing oocytes. As a result, germ cell flux is normal (Supplementary Fig. 6a) but unfertilized oocytes accumulate in the uterus and are eventually expelled from the body; no viable progeny are produced^27^. We found that germline progenitors in this mutant were lost similarly to those in wild type (Fig. 3a). Taken together, our mutant analysis suggests that germ cell flux, rather than sperm, fertilization or viable progeny production promotes germline progenitor cell loss over time.

### Germ cell flux acts partly through DAF-16/FOXO

**Figure 4:**
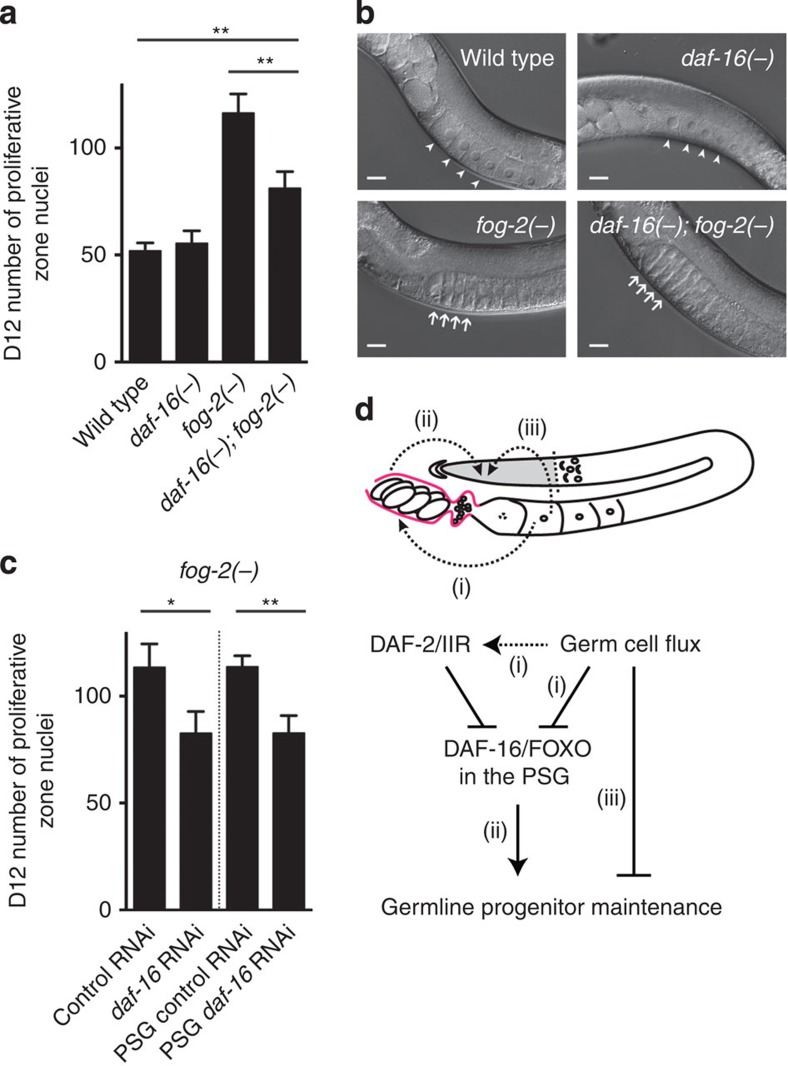
Germ cell flux influences germline progenitor maintenance through DAF-16/FOXO-dependent and DAF-16/FOXO-independent mechanisms. (**a**) Average number of proliferative zone nuclei in D12 wild-type, *daf-16(−)*, *fog-2(−)* and *daf-16(−); fog-2(−)* animals. (**b**) Representative DIC images of D1 wild-type, *daf-16(−)*, *fog-2(−)* and *daf-16(−); fog-2(−)* germ lines. Arrowhead: normal oocytes. Arrow: stacked oocytes. All *daf-16(−); fog-2(−)* animals show the oocyte stacking phenotype (*n*>50). Scale bars, 20 μm. (**c**) Average number of proliferative zone nuclei in D12 *fog-2(−)* and *rde-1(−), fog-2(−); Is[Pfos-1a::rde-1(+)]* animals treated with control and *daf-16* RNAi. Error bar indicates s.e.m.; *P<0.05; **P<0.01 by two-tailed Student's *t*-test. Alleles used are as follows: *fog-2(oz40)*, *daf-16(mu86)* and *rde-1(ne219)*. See Supplementary Table 3 for complete genotypes and data. (**d**) Model. Pink line outlines the proximal somatic gonad. Areas for future inquiry are as follows: (i) links between germ cell flux and IIS (the effects of germ cell flux that are DAF-16/FOXO dependent may occur through DAF-2/IIR, as indicated by dotted arrow); (ii) relevant DAF-16/FOXO targets in the proximal somatic gonad that signal to the distal stem/progenitor cell pool; and (iii) mechanism by which germ cell flux influences germline progenitors that is independent of proximal somatic gonad DAF-16/FOXO activity. DIC, differential interference contrast.

Since either blocking germ cell flux or reducing *daf-2* activity causes a similar phenotype in which old worms maintain a larger population of germline progenitors, and since the *daf-2(rf)* effect is suppressed by loss of *daf-16*, we asked whether the maintenance of germline progenitors in response to reduced germ cell flux is also dependent on *daf-16*. We found that in the absence of *daf-16*, *fog-2(−)* animals lost more germline progenitors over time, although not to the same extent as the wild type (Fig. 4a, *daf-16(−); fog-2(−)* versus *fog-2(−)* and wild type, respectively). This result suggests that *daf-16(+)* activity is required for the full effect of blocking germ cell flux to maintain germline progenitors. Importantly, loss of *daf-16* did not restore germ cell flux in the *fog-2(−)* animals (Fig. 4b; Supplementary Fig. 6b), ruling out the possibility that the loss of germline progenitors seen in the *daf-16(−); fog-2(−)* double mutant is simply a result of resuming germ cell flux. Consistent with a major role for *daf-16* in mediating the effect of germ cell flux on germline progenitor maintenance, loss of *daf-16* abolished the effect of mating (therefore, increasing germ cell flux) on the germline progenitor pool in the *daf-2(rf)* animals (Supplementary Fig. 7).

To determine whether *daf-16(+)* is also required in the proximal somatic gonad in this context, we performed tissue-specific *daf-16* RNAi. We found that knocking down *daf-16* in the proximal somatic gonad alone had the same effect as *daf-16* RNAi in the whole animal: more pronounced progenitor loss in the *fog-2(−)* low-flux background (Fig. 4c). Together, these data suggest that germ cell flux influences germline progenitor maintenance through mechanisms that are dependent and independent of DAF-16/FOXO activity in the proximal somatic gonad.

## Discussion

Our results show that (1) the stem/progenitor cell pool in the *C. elegans* germ line becomes depleted over time; (2) reducing IIS maintains this pool through DAF-16/FOXO activity in the proximal somatic gonad; and (3) blocking germ cell flux also maintains germline progenitors and this effect partially depends on proximal somatic gonad DAF-16/FOXO activity (Fig. 4d).

Our results offer three conceptual implications that may be generally relevant to age-related behaviour of stem cells. First, we demonstrate that the extent of germline progenitor cell loss over time can be uncoupled from the rate of organismal aging: intestinal *daf-16(+)* activity extends lifespan, but has no effect on age-dependent germline progenitor cell loss, while proximal somatic gonad *daf-16(+)* activity maintains germline progenitors without having much effect on lifespan. These results indicate that even though germline progenitor maintenance over time and lifespan are both regulated by IIS, they are independent phenotypes and DAF-16/FOXO regulates these two processes separately. We speculate that in other species, aging of tissue stem cells and organismal aging may similarly share molecular pathways but diverge in terms of regulative cell autonomy.

Although our results demonstrate distinct anatomical regulation of germline progenitor maintenance and lifespan at the level of DAF-16/FOXO activity, they may be linked upstream since both respond to DAF-2/IIR. From an evolutionary perspective, it is noteworthy that DAF-2 levels may thereby have a coordinate effect on reproduction and lifespan. For example, reducing *daf-2* activity both extends lifespan and maintains germline progenitors, the combination of which could conceivably coordinately extend reproductive span and lifespan.

Second, our results imply that maintenance of tissue stem cells is not necessarily prioritized. In contrast to a scenario where a tissue stem cell population is positively regulated by maturation of the relevant progeny cells (thereby maintaining progenitor cell numbers), we show that under laboratory conditions, germline progenitors in *C. elegans* are ‘used up'. That is, blocking germ cell flux maintains germline progenitors, while increasing germ cell flux exacerbates the loss of germline progenitors over time. This interpretation is consistent with the reduction in germline progenitors seen in wild-type animals mated over D1–D4 (ref. 28). We speculate that in this relatively short-lived species, maintenance of a large population of germline progenitors is not necessary as reproduction progresses. This idea is compatible with an evolutionary prioritization of an optimal, rather than maximal, number of progeny suggested previously by Hughes *et al*.^12^. Maintenance of other types of tissue stem cells may not be prioritized in other contexts where their maintenance may have been evolutionarily costly.

Finally, our data show that maintenance of the distal stem/progenitor cell pool can be influenced by germ cell flux or IIS output from somatic cells at the proximal end of the reproductive tract. Importantly, this proximal area contacts mature gametes and embryos that were derived from the distal stem/progenitor cell pool. Thus, in addition to signals from the local niche^2^ and the systemic environment^29^, stem/progenitor cells can be regulated at the tissue/organ level. Similar mechanisms may operate in other tissue stem cell systems.

So how could *C. elegans* maintain germline progenitors when germ cell flux is reduced? We speculate that a product generated under conditions of reduced flux, for example, from stacked oocytes or germ cells undergoing a delay in meiotic progression^30^, may relay a signal to the distal germ line that retains germline progenitors until, for example, the hermaphrodite mates and can resume active reproduction.

Our studies also raise interesting questions for future enquiry such as the precise links between germ cell flux and IIS in regulating germline progenitor maintenance, the targets of DAF-16/FOXO in the proximal somatic gonad that signal to the distal stem/progenitor cell pool, and the mechanism by which germ cell flux influences germline progenitors that is independent of proximal somatic gonad DAF-16/FOXO activity (Fig. 4d).

## Methods

### Strains and plasmids

Strains were derived from N2 wild type (Bristol) and handled using standard methods^31^. Unless otherwise indicated, worms were grown on OP50 at 20 °C. Strains and plasmids constructed for this study are as follows: GC1332 *daf-16(mu86); daf-2(e1370)*, GC1285 *daf-16(m26); daf-2(e1370); naEx239[pGC629(Pfos-1a::gfp::daf-16)+pRF4]*, GC1337 *rde-1(ne219); qyIs102[Pfos-1a::rde-1(genomic)+myo-2::yfp+unc-119(+)]*, GC1352 *daf-2(e1370); rde-1(ne219); qyIs102[Pfos-1a::rde-1(genomic)+myo-2::yfp+unc-119(+)]*, GC1335 *daf-16(mu86); fog-2(oz40)*, GC1353 *rde-1(ne219), fog-2(oz40); qyIs102[Pfos-1a::rde-1(genomic)+myo-2::yfp+unc-119(+)]* and pGC629 *Pfos-1a::gfp::daf-16*. Full genotypes of strains used are in Supplementary Table 1. Construction details for strains and plasmids are in Supplementary Tables 1 and 2, respectively.

### Analysis of germline progenitor cells

Synchronization by L1 hatch-off, ethanol fixation and 4,6-diamidino-2-phenylindole staining were performed as described^32^. For hatch-off synchronization, all larvae and adults were washed off the bacterial lawn of plates of mixed-stage worms with M9 buffer. The remaining embryos were allowed to hatch for 2 h at 20 °C. Newly hatched L1 larvae were collected, distributed to plates seeded with OP50 or RNAi bacteria and grown at 20 °C. Worms were then staged by vulval development (see Fig. 4 of ref. 33), transferred onto fresh plates every other day until reproduction cessation and fixed at 1, 6 and 12 days post-L4 stage. For fixation and staining, worms were washed off plates with M9 buffer, fixed in 100% ethanol for 5–10 min and stained using VECTASHIELD mounting medium with 4,6-diamidino-2-phenylindole (Vector Laboratories, Burlingame, CA, USA).

Microscopy and determination of number of nuclei in the proliferative zone, distance to meiotic entry and mitotic index were as described^13^. All images were acquired with a Zeiss Imager Z1 equipped with an Apotome Axioimager (Carl Zeiss). The number of proliferative zone nuclei included all the germ cell nuclei between the distal tip and the beginning of meiotic entry, defined as the first row of cells in which two or more nuclei displayed the characteristic crescent shape. Distance to meiotic entry was measured in cell diameters from the distal tip to the meiotic entry border. The mitotic index was determined as the percentage of metaphase and anaphase figures over the total number of proliferative zone nuclei.

### RNAi

RNAi by bacterial feeding was performed as described^34^: worms were synchronized by L1 hatch-off (as described above), put onto plates with HT115 carrying control (L4440) or experimental plasmid and transferred onto fresh plates every other day until reproduction cessation at 20 °C. All RNAi results were pooled from at least two independent experiments.

### Lifespan analysis

Lifespan experiments were performed at 20 °C as described^35^: ∼100 L4 (*t*_0_) larvae were picked and transferred onto fresh plates every other day until reproduction cessation. Worms that crawled off the plate or died as a result of extruded internal organs (‘bursting') or internally hatched progeny (‘bagging') were censored but were included in the statistical analysis as censored animals. Survival curves were generated using Prism 6.

### Mating

Mating experiments were performed as described^12^: day 4 adult wild-type hermaphrodites or *fog-2(oz40)* females were mated with three young, wild-type adult males individually for 2 days. Mating was later confirmed by the presence of male progeny.

### Quantitation of ovulation rate

Ovulation rate of D1 animals were determined as in ref. 36. First, worms were mounted in M9 for Nomarski microscopy and the number of oocytes and embryos in the spermathecae and uterus of each animal was determined. Animals were then transferred individually to fresh plates with OP50 for 2–3 h. At the end of the time interval, oocytes and embryos in the spermathecae and uterus were counted again. Embryos or unfertilized oocytes laid on the plate were also counted. The ovulation rate per gonad arm per hour was then calculated.

## Author contributions

Z.Q. and E.J.A.H. designed the research; Z.Q. performed the experiments; Z.Q. analysed the data; and Z.Q. and E.J.A.H. wrote the manuscript.

## Additional information

**How to cite this article**: Zhao Qin & E. Jane Albert Hubbard, Non-autonomous DAF-16/FOXO activity antagonizes age-related loss of *C. elegans* germline stem/progenitor cells. *Nat. Commun*. 6:7107 doi: 10.1038/ncomms8107 (2015).

## Supplementary Material

Supplementary Information Supplementary Figures 1-7, Supplementary Tables 1-4 and Supplementary References
