# Supplementary material for: Non-autonomous DAF-16/FOXO activity antagonizes age-related loss of C. elegans germline stem/progenitor cells
Source: Nat Commun. 2015 May 11;6:7107. doi: 10.1038/ncomms8107 (PMC4432587; doi:10.1038/ncomms8107)
Supplement: Supplementary Information — Supplementary Figures 1-7, Supplementary Tables 1-4 and Supplementary References [file ncomms8107-s1.pdf]

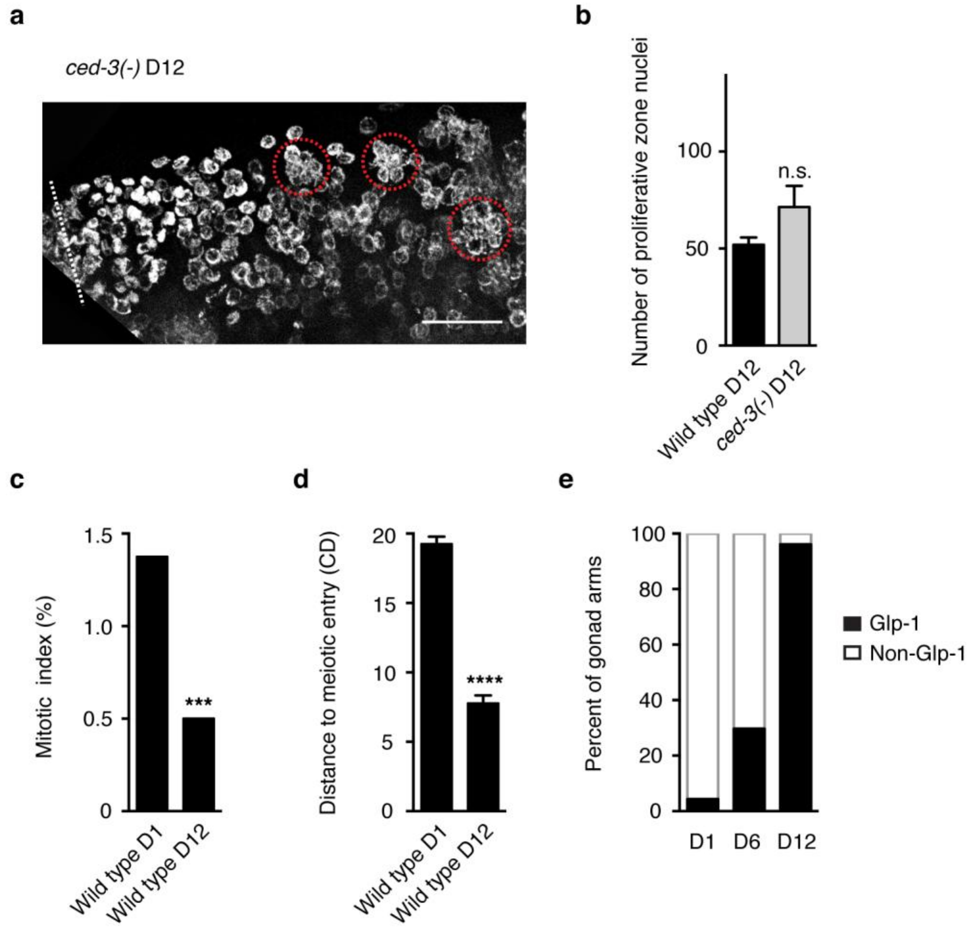

Supplementary Figure 1. Both cell cycle and cell fate decision, but not apoptosis, contribute to the loss of germline progenitors over time. (a) Representative DAPI-stained *ced-3(-)* germ line. Dotted line indicates the proximal boarder of the proliferative zone. Red dotted circles indicate clusters of extra germ cells accumulated in the pachytene stage. Scale bar: 20 μm. (b) Average number of proliferative zone nuclei in D12 wild-type and *ced-3(-)*animals. Error bar indicates s.e.m.;n.s., *p*> 0.05 by two-tailed Student's *t*-test. (c) Mitotic index of D1 and D12 wild-type germline proliferative zone. The number ofgonad arms and number of germ cells analyzed: 33 and 6469 for D1, 23 and

1195 for D12. ***, $p < 0.001$ by Mann-Whitney U test. (d) Distance in cell diameters (CD) from the distal tip to the proximal boarder of the proliferative zone in D1 and D12 wild-type animals. Same animals were analyzed as in (c). Error bar indicatess.e.m.; ****, $p < 0.0001$ by two-tailed Student's *t*-test. (e) Percent of *glp-1(rf)* gonad arms scored at D1, D6, and D12 showing the 'all meiotic' Glp-1 (black) and non-Glp-1 (white) phenotypes. n> 20 for each time point. Alleles used: *ced-3(n717)* and *glp-1(e2141).*See Supplementary Table 3 for complete data.

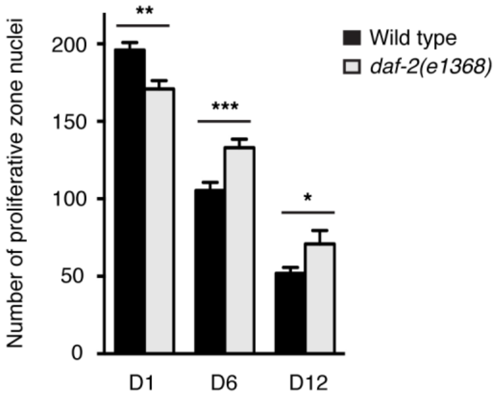

Supplementary Figure 2.Germline progenitor cell depletion is attenuated in*daf-2(e1368)* animals. Error bar indicates s.e.m.;*, $p < 0.05$; **, $p < 0.01$; ***, $p < 0.001$ by two-tailed Student's *t*-test; also, pairwise comparisons within genotypes for each time point $p < 0.0001$ by Student's *t*-test. See Supplementary Table 3 for complete data.

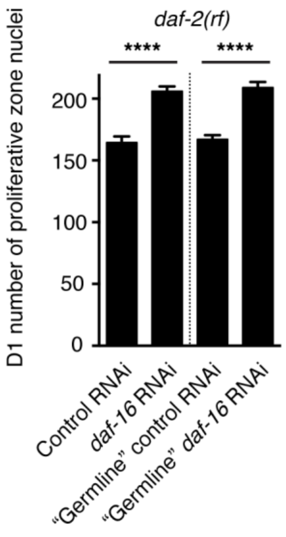

Supplementary Figure 3.Germline DAF-16/FOXO activity inhibits larval germline proliferation. Average number of proliferative zone nuclei in D1*daf-2(rf)* and *rrf-1(-); daf-2(rf)* animals treated with control and *daf-16*RNAi. Error bar indicates s.e.m.; ****, $p < 0.0001$ by two-tailed Student's *t*-test.Alleles used: *daf-2(e1370)*and *rrf-1(pk1417)*.See Supplementary Table 3 for complete data.

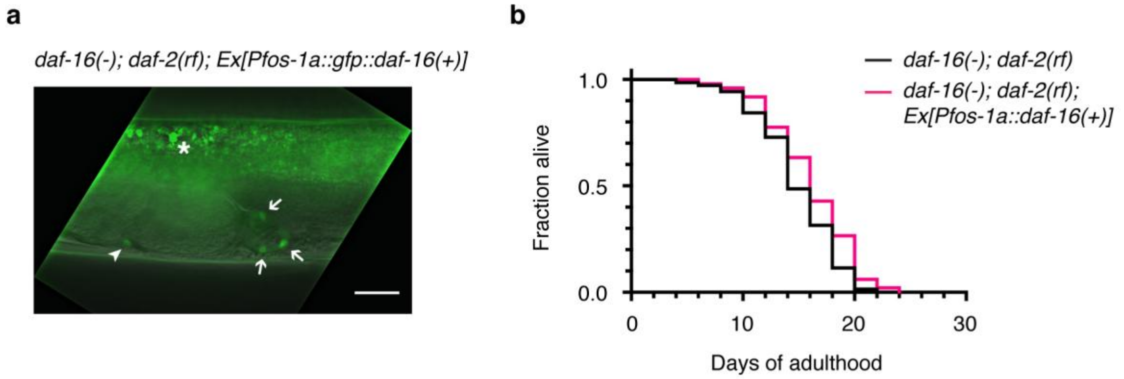

Supplementary Figure 4. Characterization of *Pfos-1a::gfp::daf-16(+)*. (a) Expression of *gfp::daf-16(+)*inthe spermatheca (arrow) and uterus(arrowhead). Note that GFP::DAF-16 is localized to the nucleus in the *daf-16(-); daf-2(rf)*background. Asterisk indicates autofluorescence in the intestine. Scale bar: 20 µm. (b) Survival curve of *daf-16(-); daf-2(rf); Ex[Pfos-1a::gfp::daf-16(+)]* animals and their non-transgenic siblings. $p = 0.04$ by Mantel-Cox log-rank test. Alleles used: *daf-2(e1370)* and *daf-16(m26)*.See Supplementary Table 4 for complete genotypes and data.

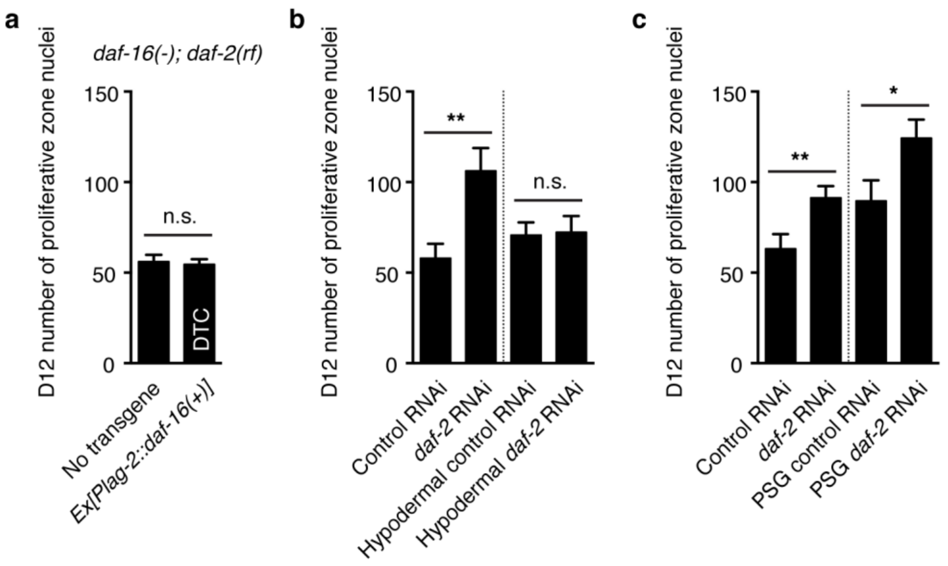

Supplementary Figure 5. IIS is required for germline progenitor maintenance over time in the proximal somatic gonad (PSG), but not in the distal tip cell (DTC) or hypodermis. Average number of proliferative zone nuclei in D12 (a) *daf-16(-); daf-2(rf)*and *daf-16(-); daf-2(rf)* animals carrying a transgene expressing *daf-16(+)::gfp* from the*lag-2* promoter, (b)*Is(Plin-26::nls::gfp)*and *rde-1(-); Is[Plin-26::nls::gfp + Plin-26::rde-1(+)]*animals treated with control and *daf-2*RNAi, (c) wild-type and *rde-1(-); Is[Pfos-1a::rde-1(+)]*animals treated with control and *daf-2*RNAi. (a) Label on the bar indicates the relevant tissue that expresses the transgene. Error bar indicates s.e.m.;n.s., $p > 0.05$; *, $p < 0.05$; **, $p < 0.01$ by two-tailed Student's *t*-test. Allelesused: *daf-2(e1370)*, *daf-16(m26)*, and *rde-1(ne219)*. See Supplementary Table 3 for complete genotypes and data.

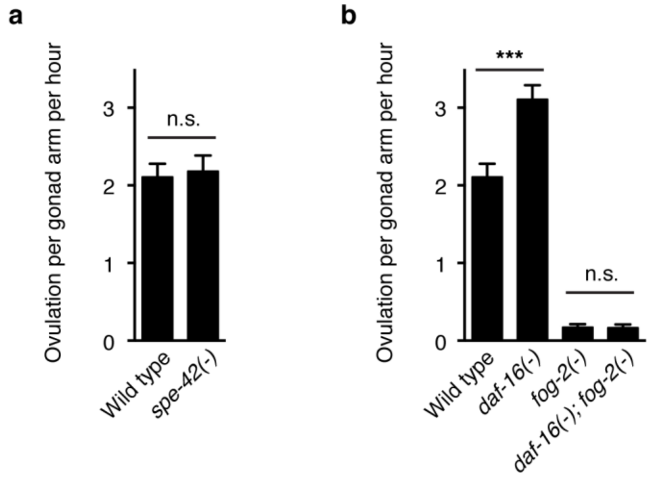

Supplementary Figure 6.Germ cell flux in *spe-42(-)*is comparable to that in wild type, and loss of *daf-16* does not restore germ cell flux in *fog-2(-)* animals. Average ovulation rate per gonad arm per hour in D1 (a) wild type and *spe-42(-)*mutants, (b) wild-type, *daf-16(-), fog-2(-),*and *daf-16(-); fog-2(-)*animals. Error bar indicates s.e.m.;n.s., $p > 0.05$; ***, $p < 0.001$ by two-tailed Student's *t*-test. n> 10 for each genotype.Allelesused: *spe-42(tn1231), daf-16(mu86)*, and *fog-2(oz40).*

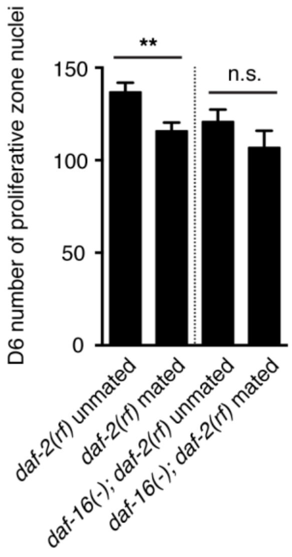

Supplementary Figure 7.Loss of *daf-16* abolishes the effect of increasing germ cell flux on germline progenitors in the *daf-2(rf)* mutants. Average number of proliferative zone nuclei in D6 *daf-2(rf)* and *daf-16(-); daf-2(rf)* animals that are unmated or have been mated with young, wild-type adult males since D4. Note that the effect of increasing germ cell flux by mating on *daf-2(rf)* animals is not as strong as that on wild type and *fog-2(-)* (see Fig. 3b), probably due to thefact that*daf-2(rf)* mutantshave an extended reproductive span and produce more progeny than unmated wild-type and *fog-2(-)* animals from D4 to D6. Therefore during this time interval, germ cell flux in the *daf-2(rf)*unmated control is higher. Error bar indicates s.e.m.;n.s., $p > 0.05$; **, $p < 0.01$ by two-tailed Student's *t*-test. Alleles used: *daf-2(e1370)* and *daf-16(mu86)*. See Supplementary Table 3 for complete genotypes and data.

Supplementary Tables

Supplementary Table 1. Strains.

**Strain**	**Genotype**	**Reference/Source (see Supplementary References)**
N2	wild type	[1]
MT1522	*ced-3(n717)*	[2]
CB4037	*glp-1(e2141)*	[3]; Dalfo*et al.*, 2010 Wormbreeder's Gazette Vol 18 no 3; http://www.wormbook.org/wbg/articles/volume-18-number-3/glp-1e2141-sequence-correction/
CB1370	*daf-2(e1370)*	[4]
DR1572	*daf-2(e1368)*	[5]
CF1038	*daf-16(mu86)*	[6]
GC1332	*daf-16(mu86); daf-2(e1370)*	This work: made by losing extrachromosomal array from CF1449
CF1514	*daf-16(mu86); daf-2(e1370); muEx211[Pges-1::gfp::daf-16 + pRF4]*	[7]
CF1515	*daf-16(mu86); daf-2(e1370); muEx212[Pmyo-3::gfp::daf-16 + pRF4]*	[7]
CF1442	*daf-16(mu86); daf-2(e1370); muEx169[Punc-119::gfp::daf-16 + pRF4]*	[7]
CF1449	*daf-16(mu86); daf-2(e1370); muEx176[Pdaf-16::gfp::daf-16 + pRF4]*	[7]
DR1309	*daf-16(m26); daf-2(e1370)*	P. Albert and D. Riddle, via CGC
GC1285	*daf-16(m26); daf-2(e1370); naEx239[pGC629(Pfos-1a::gfp::daf-16) + pRF4]*	This work: pGC629 (1 ng/µl) was injected to DR1309 with pRF4 (100 ng/µl).
GC1109	*daf-16(m26); daf-2(e1370); naEx202[pGC461(Plag-2::daf-16::gfp) + pRF4]*	[8]
GC1019	*rrf-1(pk1417); daf-2(e1370)*	[8]
GC1337	*rde-1(ne219); qyIs102[Pfos-1a::rde-1(genomic) + myo-2::yfp + unc-119(+)]*	This work: *rrf-3(pk1426)* was crossed out from NK640[9]
GC1352	*daf-2(e1370); rde-1(ne219); qyIs102[Pfos-1a::rde-1(genomic) + myo-2::yfp + unc-119(+)]*	This work: GC1337 was crossed with CB1370

NR220	*kzIs7(Plin-26::nls::gfp + pRF4)*	[10]
NR222	*rde-1(ne219); kzIs9[pKK1260(Plin-26::nls::gfp) + pKK1253(Plin-26::rde-1) + pRF4]*	[10]
BS553	*fog-2(oz40)*	[11]
DG2347	*acy-4(ok1806); tnEx37(acy-4(+) + sur-5::gfp)*	[12]
MT2136	*lin-3(n1058)/unc-8(e49) dpy-20(e1362)*	[13]
SL1138	*spe-42(tn1231)/nT1 [unc-?(n754) let-? qIs50]*	[14]
GC1335	*daf-16(mu86); fog-2(oz40)*	This work: CF1038 was crossed with BS553
GC1353	*rde-1(ne219), fog-2(oz40); qyIs102[Pfos-1a::rde-1(genomic) + myo-2::yfp + unc-119(+)]*	This work: GC1337 was crossed with BS553

Supplementary Table 2. Plasmids.

**Plasmid**	**Description**	**Reference/Construction**
sjj_R13H8.2	*daf-16(RNAi)*	[15]
pGC488	*daf-2(RNAi)*	[8]
pGC629	*Pfos-1a::gfp::daf-16*	*fos1a* promoter was PCR amplified from pBS-fos-1p (a kind gift from David Sherwood) using 5'-CGGAATTAACCCTCACTAAAGGGAACAAAAGCTGGAGTACgcccaactctagtcatttct-3' and 5'-GTACCGCCCCCTCGAGGTCGACGGTATCGATAAGCTTTACtccactctcttatatagcag-3', and inserted into pNL205[7]cut by SnaBI using Gibson assembly.

Supplementary Table 3. Number of proliferative zone nuclei.

Strain	Genotype	Treatment	Stage	Average	s.e.m.	n
**Fig. 1c**						
N2	wild type		D1	196.0	±4.8	33
CB1370	*daf-2(e1370)*		D1	160.8	±6.0	22
N2	wild type		D6	105.3	±5.3	25
CB1370	*daf-2(e1370)*		D6	136.9	±5.0	22
N2	wild type		D12	52.0	±3.7	23
CB1370	*daf-2(e1370)*		D12	112.0	±6.8	22
**Fig. 2a**						
N2	wild type		D12	52.0	±3.7	23
CF1038	*daf-16(mu86)*		D12	55.5	±5.8	23
CB1370	*daf-2(e1370)*		D12	112.0	±6.8	22
GC1332	*daf-16(mu86); daf-2(e1370)*		D12	54.0	±8.4	16
**Fig. 2b**						
GC1332	*daf-16(mu86); daf-2(e1370)*		D12	54.0	±8.4	16
CF1514	*daf-16(mu86); daf-2(e1370); muEx211[Pges-1::gfp::daf-16 + pRF4]*		D12	59.1	±4.5	20
CF1515	*daf-16(mu86); daf-2(e1370); muEx212[Pmyo-3::gfp::daf-16 + pRF4]*		D12	49.7	±3.7	23
CF1442	*daf-16(mu86); daf-2(e1370); muEx169[Punc-119::gfp::daf-16 + pRF4]*		D12	53.9	±3.9	23
CF1449	*daf-16(mu86); daf-2(e1370); muEx176[Pdaf-16::gfp::daf-16 + pRF4]*		D12	102.3	±9.0	22
**Fig. 2c**						
DR1309	*daf-16(m26); daf-2(e1370)*		D12	56.0	±3.8	23
GC1285	*daf-16(m26); daf-2(e1370); naEx239[pGC629(Pfos-1a::gfp::daf-16) + pRF4]*		D12	103.5	±8.9	22
**Fig. 2d**						

CB1370	*daf-2(e1370)*	controlRNAi	D12	109.2	±4.5	24
CB1370	*daf-2(e1370)*	*daf-16* RNAi	D12	79.1	±6.0	20
GC1019	*rrf-1(pk1417); daf-2(e1370)*	controlRNAi	D12	108.6	±5.2	25
GC1019	*rrf-1(pk1417); daf-2(e1370)*	*daf-16*RNAi	D12	153.3	±8.2	25
**Fig. 2e**						
CB1370	*daf-2(e1370)*	controlRNAi	D12	109.2	±4.5	24
CB1370	*daf-2(e1370)*	*daf-16* RNAi	D12	79.1	±6.0	20
GC1352	*daf-2(e1370); rde-1(ne219); qyIs102[Pfos-1a::rde-1(genomic) + myo-2::yfp + unc-119(+)]*	controlRNAi	D12	103.5	±5.5	20
GC1352	*daf-2(e1370); rde-1(ne219); qyIs102[Pfos-1a::rde-1(genomic) + myo-2::yfp + unc-119(+)]*	*daf-16*RNAi	D12	79.4	±6.7	23
**Fig. 3a**						
N2	wild type		D12	52.0	±3.7	23
BS553	*fog-2(oz40)*		D12	116.3	±9.0	24
Progeny of DG2347	*acy-4(ok1806)*		D12	120.3	±14.2	6
Progeny of MT2136	*lin-3(n1058)*		D12	125.5	±18.9	11
Progeny of SL1138	*spe-42(tn1231)*		D12	45.8	±4.0	14
**Fig. 3b**						
N2	wild type	unmated	D6	105.3	±5.3	25
N2	wild type	mated	D6	57.3	±5.1	24
BS553	*fog-2(oz40)*	unmated	D6	154.6	±8.1	22
BS553	*fog-2(oz40)*	mated	D6	116.0	±7.8	23
**Fig. 4a**						
N2	wild type		D12	52.0	±3.7	23
CF1038	*daf-16(mu86)*		D12	55.5	±5.8	23
BS553	*fog-2(oz40)*		D12	116.3	±9.0	24
GC1335	*daf-16(mu86); fog-2(oz40)*		D12	81.2	±7.8	25
**Fig. 4c**						
BS553	*fog-2(oz40)*	controlRNAi	D12	113.5	±10.9	14

BS553	*fog-2(oz40)*	*daf-16* RNAi	D12	82.6	±10.2	17
GC1353	*rde-1(ne219), fog-2(oz40); qyIs102[Pfos-1a::rde-1(genomic) + myo-2::yfp + unc-119(+)]*	controlRNAi	D12	113.7	±5.2	22
GC1353	*rde-1(ne219), fog-2(oz40); qyIs102[Pfos-1a::rde-1(genomic) + myo-2::yfp + unc-119(+)]*	*daf-16*RNAi	D12	82.7	±8.2	20
**Supplementary Fig. 1a**						
N2	wild type		D12	52.0	±3.7	23
MT1522	*ced-3(n717)*		D12	71.4	±10.9	21
**Supplementary Fig. 2**						
N2	wild type		D1	196.0	±4.8	33
DR1572	*daf-2(e1368)*		D1	171.0	±5.3	20
N2	wild type		D6	105.3	±5.3	25
DR1572	*daf-2(e1368)*		D6	133.0	±5.5	21
N2	wild type		D12	52.0	±3.7	23
DR1572	*daf-2(e1368)*		D12	70.9	±8.7	20
**Supplementary Fig. 3**						
CB1370	*daf-2(e1370)*	controlRNAi	D1	164.4	±5.0	22
CB1370	*daf-2(e1370)*	*daf-16* RNAi	D1	206.0	±3.9	25
GC1019	*rrf-1(pk1417); daf-2(e1370)*	controlRNAi	D1	167.0	±3.4	24
GC1019	*rrf-1(pk1417); daf-2(e1370)*	*daf-16*RNAi	D1	208.9	±4.5	28
**Supplementary Fig. 5a**						
DR1309	*daf-16(m26); daf-2(e1370)*		D12	56.0	±3.8	23
GC1109	*daf-16(m26); daf-2(e1370); naEx202[pGC461(Plag-2::daf-16::gfp) + pRF4]*		D12	54.5	±2.9	22
**Supplementary Fig. 5b**						
NR220	*kzIs7(Plin-26::nls::gfp + pRF4)*	controlRNAi	D12	58.0	±7.9	20
NR220	*kzIs7(Plin-26::nls::gfp + pRF4)*	*daf-2* RNAi	D12	106.2	±12.6	17
NR222	*rde-1(ne219); kzIs9[pKK1260(Plin-26::nls::gfp) + pKK1253(Plin-26::rde-1) + pRF4]*	controlRNAi	D12	70.8	±7.0	22

NR222	*rde-1(ne219); kzIs9[pKK1260(Plin-26::nls::gfp) + pKK1253(Plin-26::rde-1) + pRF4]*	*daf*-2RNAi	D12	72.3	±9.0	24
**Supplementary Fig. 5c**						
N2	wild type	controlRNAi	D12	63.2	±8.1	20
N2	wild type	*daf*-2RNAi	D12	91.4	±6.4	21
GC1337	*rde-1(ne219); qyIs102[Pfos-1a::rde-1(genomic) + myo-2::yfp + unc-119(+)]*	controlRNAi	D12	89.6	±11.4	12
GC1337	*rde-1(ne219); qyIs102[Pfos-1a::rde-1(genomic) + myo-2::yfp + unc-119(+)]*	*daf*-2RNAi	D12	124.3	±10.3	15
**Supplementary Fig. 7**						
CB1370	*daf-2(e1370)*	unmated	D6	136.9	±5.0	22
CB1370	*daf-2(e1370)*	mated	D6	115.9	±4.5	14
GC1332	*daf-16(mu86); daf-2(e1370)*	unmated	D6	120.9	±6.6	20
GC1332	*daf-16(mu86); daf-2(e1370)*	mated	D6	106.8	±9.1	13

Supplementary Table 4. Lifespan data of *daf-16(-); daf-2(rf); Ex[Pfos-1a::gfp::daf-16(+)]*.

**Strain**	**Genotype**	**Mean lifespan ± s.e.m.(days)**	**Observed/ Total**	**Change**	***P***
Progeny of GC1285	*daf-16(-); daf-2(rf)*	14.8±0.4	70/100	-	-
Progeny of GC1285	*daf-16(-); daf-2(rf); Ex[Pfos-1a::gfp::daf-16(+)]*	16.1±0.5	49/100	8.7%	0.04

References for SupplementaryInformation

1. Brenner, S. The genetics of *Caenorhabditis elegans*. *Genetics***77,** 71–94 (1974).

2. Gumienny, T. L., Lambie, E., Hartwieg, E., Horvitz, H. R. & Hengartner, M. O. Genetic control of programmed cell death in the *Caenorhabditis elegans* hermaphrodite germline. *Development***126,** 1011–1022 (1999).

3. Priess, J. R., Schnabel, H. & Schnabel, R. The *glp-1* locus and cellular interactions in early *C. elegans* embryos. *Cell***51,** 601–611 (1987).

4. Riddle, D. L., Swanson, M. M. & Albert, P. S. Interacting genes in nematode dauer larva formation. *Nature***290,** 668–671 (1981).

5. Gems, D. *et al.* Two pleiotropic classes of *daf-2* mutation affect larval arrest, adult behavior, reproduction and longevity in *Caenorhabditis elegans*. *Genetics***150,** 129–155 (1998).

6. Lin, K., Dorman, J. B., Rodan, A. & Kenyon, C. *daf-16*: An HNF-3/forkhead family member that can function to double the life-span of *Caenorhabditis elegans*. *Science***278,** 1319–1322 (1997).

7. Libina, N., Berman, J. R. & Kenyon, C. Tissue-specific activities of *C. elegans* DAF-16 in the regulation of lifespan. *Cell***115,** 489–502 (2003).

8. Michaelson, D., Korta, D. Z., Capua, Y. & Hubbard, E. J. A. Insulin signaling promotes germline proliferation in *C. elegans*. *Development***137,** 671–680 (2010).

9. Hagedorn, E. J. *et al.* Integrin acts upstream of netrin signaling to regulate formation of the anchor cell's invasive membrane in *C. elegans*. *Dev Cell***17,** 187–

198 (2009).

10. Qadota, H. *et al.* Establishment of a tissue-specific RNAi system in *C. elegans*. *Gene***400,** 166–173 (2007).

11. Clifford, R. *et al.* FOG-2, a novel F-box containing protein, associates with the GLD-1 RNA binding protein and directs male sex determination in the *C. elegans* hermaphrodite germline. *Development***127,** 5265–5276 (2000).

12. Govindan, J. A., Nadarajan, S., Kim, S., Starich, T. A. & Greenstein, D. Somatic cAMP signaling regulates MSP-dependent oocyte growth and meiotic maturation in *C. elegans*. *Development***136,** 2211–2221 (2009).

13. Clandinin, T. R., DeModena, J. A. & Sternberg, P. W. Inositol trisphosphate mediates a RAS-independent response to LET-23 receptor tyrosine kinase activation in *C. elegans*. *Cell***92,** 523–533 (1998).

14. Kroft, T. L., Gleason, E. J. & L'Hernault, S. W. The *spe-42* gene is required for sperm-egg interactions during *C. elegans* fertilization and encodes a sperm-specific transmembrane protein. *Dev. Biol.***286,** 169–181 (2005).

15. Kamath, R. S. *et al.* Systematic functional analysis of the *Caenorhabditis elegans* genome using RNAi. *Nature***421,** 231–237 (2003).
